# Influence of UV-C Irradiation Duration on Seed-Borne Fungal Suppression, Germination, and Seedling Development in Rice (*Oryza sativa* L.)

**DOI:** 10.3390/biology15120957

**Published:** 2026-06-18

**Authors:** Saleh M. Al-Sager, Fayza H. Gomaa, Sherihan M. M. Bekheet, Waleed A. Almasoud, Saleh Al-Ghamdi, Saad S. Almady, Abdulwahed M. Aboukarima, Mohamed E. Yehia

**Affiliations:** 1 Department of Agricultural Engineering, College of Food and Agriculture Sciences, King Saud University, P.O. Box 2460, Riyadh 11451, Saudi Arabia; salsaqer@ksu.edu.sa (S.M.A.-S.); almasoud@ksu.edu.sa (W.A.A.); sasaleh@ksu.edu.sa (S.A.-G.); salmady@ksu.edu.sa (S.S.A.); 2 Seed Pathology Research Department, Plant Pathology Research Institute, Agriculture Research Center, Giza 12619, Egypt; fayzahassan2003@yahoo.com; 3 Rice Technology Training Centre, Field Crops Research Institute, Agricultural Research Center, Giza 12619, Egypt; mohamed_yehia_eg@yahoo.com

**Keywords:** Bio-sanitization, rice seeds, seedling vigor, UV radiation

## Abstract

The present study was conducted to study the effect of exposure time to ultraviolet-C (UV-C) radiation on seed germination, fungal suppression and seedling growth of three Egyptian rice cultivars, namely, Sakha 105, Sakha 108, and Giza 183. Experiments were carried out under controlled laboratory conditions. Rice seeds were exposed to UV-C radiation with a wavelength of 253.7 nm and intensity of 1960 µW cm^2^ for 0 (control), 10, 20, 30, 40, 50, and 60 min. Initial seed health testing showed the presence of several seed-borne fungi, mainly *Alternaria alternata, Rhizoctonia solani*, and *Fusarium verticillioides*, in addition to Aspergillus niger and *Aspergillus flavus*. Results revealed that UV-C exposure time, rice cultivar and their interactions significantly (*p* < 0.05) affected germination percentage, reduction percentage of seed fungal infection, and seedling growth parameters. The optimum exposure time was 30 min, which was found to maximize germination and improve shoot and root growth to achieve high levels of fungal suppression. Giza 183 exhibited the highest average germination percentage (92.40%), while Sakha 105 obtained the highest shoot height (17.00 cm) and root length (12.91 cm). The results indicate that UV-C irradiation is an effective, residue-free and environmentally sustainable seed treatment technology for improving rice seed quality as well as early seedling performance.

## Simple Summary

Rice seed transmission of fungal contaminants is a common problem that leads to poor germination and seedling vigor. Chemical treatment of seed-borne fungi is the usual practice, but the use of such chemicals is associated with environmental and health concerns. This study investigated the use of ultraviolet-C (UV-C) radiation as a green alternative for enhancing rice seed quality. The effect of UV-C irradiation on seed germination, seedling growth and fungal infection of three Egyptian rice cultivars (Sakha 105, Sakha 108 and Giza 183) was studied. Seeds were treated with different time intervals of UV-C irradiation. Results showed that UV-C treatment significantly reduced fungal contamination and improved seed germination and seedling growth. The highest germination, seedling vigor and fungal suppression compared to untreated seeds was observed at a 30 min exposure time. Longer exposure times required more energy but did not show any additional benefits. The results suggest that UV-C irradiation is a safe, chemical-free and residue-free technology, which can improve seed health and vigor with a reduction in synthetic fungicides. This approach provides a sustainable and practical strategy for the improvement of rice seed quality and for the promotion of environmentally friendly crop production.

## 1. Introduction

Seed priming techniques employing physical, chemical, or biological treatments are used to synchronize crop germination and protect plants from environmental stress. Physical treatment methods such as thermal, electromagnetic, and optical processing are used to improve seed germination and plant growth by inducing positive physiological and biochemical changes with minimal environmental consequences. Electromagnetic radiation, especially ultraviolet (UV) radiation, is one of the most extensively studied physical treatments [1]. UV radiation is the electromagnetic radiation with a wavelength (100–400 nm) longer than that of X-rays (<100 nm) but shorter than visible light (400–700 nm), as reported in [2]. Vázquez and Hanslmeier [2] classified UV irradiation into four separate spectral regions, namely vacuum UV (100–200 nm), UV-C (200–280 nm), UV-B (280–315 nm), and UV-A (315–400 nm).

UV-C radiation has attracted considerable attention because of its strong photochemical activity and lethal effects on microorganisms. Seed pre-treatment with ultraviolet radiation has emerged as a promising “green technology” for enhancing seed germination and plant development, owing to its ecological safety and cost-effectiveness. UV-C irradiation, in particular, has been widely studied for its ability to improve seed vigor while simultaneously reducing microbial contamination [3,4]. UV-C irradiation can also play a role in sustainable agricultural production through improved seed vigor, rapid germination, and improved seedling establishment, which may reduce the use of agrochemicals during the early stages of crop growth. It also controls seed-borne infections without creating the resistance to chemicals accompanying frequent use of pesticides due to its antimicrobial properties. UV-C treatment can also be integrated into organic and ecological farming systems because it is a physical rather than a chemical seed sanitization technique [5,6]. These benefits have been found due to increasing awareness of UV-C seed treatment as a sustainable and environmentally friendly way to improve agricultural productivity with less environmental impact.

Pre-sowing treatment of grain crop seeds with ultraviolet (UV) radiation—particularly UV-C and UV-A—enhances germination energy, increases sprout vigor, and improves early growth, acting as an environmentally friendly stimulant. Optimal UV exposure breaks down seed coats for faster water absorption, enhances metabolism, and disinfects seeds from pathogens [7].

Several studies have demonstrated the positive effect of UV-C irradiation on seed development and germination characteristics [8]. The main mechanism includes damage of microbial DNA, resulting in deactivation and, eventually, cell death [9]. Cross-protection is a common phenomenon in both prokaryotic and eukaryotic organisms. The response to UV radiation and temperature appears to share similar adaptive properties involving stress signaling pathways in fungal pathogens by coordinating the regulation of the trehalose synthase gene under different environmental challenges. This phenomenon may arise from the presence of alternate protective responses that use comparable physiological processes to produce resistance to the same or different stresses [10]. In addition to direct cellular damage, UV-C irradiation mainly affects microorganisms through the induction of DNA photolesions, especially cyclobutane pyrimidine dimers and 6–4 photoproducts, which can interfere with DNA replication and transcription. Thus, fungal survival after UV exposure is highly dependent on the capacity for DNA repair; particularly, photoreactivation mediated by photolyases and nucleotide excision repair [11,12,13] mechanisms can decrease the UV-induced damage and explain the variations in fungal tolerance or sensitivity to UV-C treatment. In addition to its antimicrobial properties, UV-C treatment was also found to stimulate the antioxidant defense system of plants. Grain UV-C treatment enhanced wheat seedlings’ resistance to leaf rust and powdery mildew, suggesting its potential use in integrated disease management [14]. Irradiation of rice seeds with UV light reduced microbial contamination of field-harvested grains while maintaining seed viability and stimulating plant growth [15]. Kamel et al. [16] found that the optimal exposure time for the preservation of Apiaceae spice seeds’ vitality was in the range of 25–30 min, whereas Czarnek et al. [17] found that the sterilization of wheat seedlings with UV-C light for 30 min efficiently lowered infection risks during germination.

One of the critical factors when using UV-C as an environmentally safe method for controlling seed fungal infestation is determining the appropriate dose and exposure time, as excessive exposure can weaken seed viability and negatively affect germination. UV radiation treatment of rice seeds with 8.32 kJ/m^2^ for three hours before soaking and 60 min after emergence resulted in significant improvement in seedling growth in comparison with untreated controls [18]. Moderate exposure of maize seeds to 254 nm UV-C radiation for 30–60 min led to an improved germination rates and a significant increase in the germination index [19]. Amaranth seeds exposed to UV-C for 30 min achieved the highest germination percentage and vigor, while shorter exposures (10 min) yielded the lowest rates [4]. Overall, these results suggest that UV-C irradiation has a dual effect on (i) decreasing microbial contamination through DNA damage and sterilization and (ii) enhancing physiological processes that enhance seed vigor, the germination rate and seedling growth. While the optimal duration and intensity of exposure vary by species, moderate treatments (generally 25–60 min) have consistently positive effects. This makes UV-C seed treatment an inexpensive and environmentally friendly tool for the establishment and resilience of crops.

Seed-borne diseases represent one of the most significant constraints to rice production, leading to both quantitative and qualitative yield losses [20]. There are over fifty seed-borne diseases that can affect rice crops [21]. These diseases are among the several causes of the rice crop’s poor production and losses. Abdelmoneim et al. [22] reported 16 fungal species in six rice varieties by the blotter method. El Morsy et al. [23] reported that the most common pathogens in infected seeds were *Drechslera oryzae*, *Trichoconiella padwickii*, *Pyricularia oryzae*, and *Sarocladium oryzae*. Ahmed et al. [24] reported nine seed-borne fungi in Bangladesh, which included *Fusarium oxysporum*, *F. moniliforme*, *Bipolaris oryzae*, *Alternaria padwickii*, *Curvularia lunata*, *Aspergillus flavus*, *A. niger*, *Penicillium* sp., and *Nigrospora oryzae*.

Further, investigations revealed that *Alternaria*, *Bipolaris*, *Cladosporium*, and *Fusarium* make up the majority of the fungal populations found in rice [25,26,27]. More recently, Ackaah et al. [28] demonstrated that seed discoloration is strongly linked to fungal infection: dark brown/brown seeds were associated with *Bipolaris* sp., *Fusarium* sp., *Macrophomina phaseolina*, and *Aspergillus* sp.; yellow/pale yellow seeds with *Bipolaris* sp., *Curvularia* sp., and *Botryodiplodia* sp.; and greyish-white seeds with *Aspergillus and Alternaria* sp. Fungal communities associated with rice cultivation vary significantly depending on the cultivar and geographic region.

Rice (*Oryza sativa* L.) is recognized and documented as the second most productive cereal crop worldwide. In Egypt, rice serves as a staple food and constitutes a significant portion of the daily diet [29]. Rice is one of the most significant agricultural products for farmers’ income in Egypt, where it is regarded as the second most important cereal after wheat. The total cultivated area of rice in Egypt is about 1.149 million ha, with a national average output of 3.741 tons/feddan (about 8.91 tons/ha) and a total production of 4.32 million tons of paddy rice [30]. Despite its strategic value, Egyptian rice cultivation is under severe water scarcity, and, therefore, the cultivated areas are strictly regulated by the government. Thus, improving water-use efficiency and seed quality through advanced priming have become an economic necessity. Seed treatment optimizes yields and resource efficiency, thereby compensating for the economic risk imposed by the limited water quota and rising costs of production [31,32].

Despite the literature documenting the broad-spectrum antimicrobial activity of ultraviolet radiation, seed quality and pathogen eradication are typically treated as independent parameters with little regard for the delicate compromise between physical disinfection and seed viability. Additionally, the structural, biochemical and hull-thickness differences amongst regional rice germplasms suggest that generalized UV-C treatments cannot be used worldwide. The novelty of this work is in its three-dimensional narrow approach: (1) three economically important Egyptian rice cultivars (Sakha 105, Sakha 108, and Giza 183) with different seed-coat profiles are evaluated, (2) an accurate mathematical optimization of exposure duration thresholds is defined, and (3) a simultaneous physiological/phytosanitary evaluation is provided. This work maps the exact operating boundaries where maximum sanitation is obtained without leading to phytotoxic regressions by the combined analysis of fungal pathogen suppression and explicit seedling morphological growth and seed vigor measures.

By suggesting UV-C treatment as a practical substitute for chemical fungicides, which lessens the negative effects on the environment and possible health hazards, the work also supports sustainable agriculture. Additionally, the results offer useful information for pre-sowing seed treatment procedures that can be applied in systems for processing and storing seeds. Overall, by combining microbial control with crop performance, this study enhances the use of UV-C technology in agriculture and provides a practically useful and scientifically sound way to improve the quality of rice seeds. Hence, the aim of this study was to investigate the effect of duration of exposure to UV-C radiation on seed germination, seedling growth parameters, and fungal disinfection of three main Egyptian rice cultivars (Sakha 105, Sakha 108, and Giza 183). It was postulated that moderate durations of UV-C irradiation would significantly inhibit seed-borne fungal pathogens and stimulate early seedling development without affecting seed viability, but that cultivar-dependent variations in seed structural or biochemical traits would influence sensitivity to prolonged exposure thresholds. In this work, a single UV-C intensity was utilized to separate the influence of exposure period on rice seed germination and early seedling growth. Therefore, the UV-C dose was altered by altering the exposure period rather than altering the irradiation intensity.

## 2. Materials and Methods

### 2.1. Rice Sample Collection and Preparation

Samples of rice grains were gathered from the Rice Research and Training Center, Agricultural Research Center, located in the Sakha region, Kafr Elsheikh Governorate, Egypt. Seeds from three cultivars (Sakha 105, Sakha 108, and Giza 183) were collected. The selected rice cultivars are characterized by high productivity and short growth cycles. Their strategic importance in Egypt lies in the optimal use of water consumption (saving about 20–30% versus traditional varieties) and their high resistance to blast disease [33,34].

One sample per variety (each weighing about 2.0 kg) was randomly selected from rice experiment trials in accordance with the International Seed Testing Association (ISTA) [35] regulations. Testing samples were cleaned, labeled properly, sealed in polythene bags and stored at 5 °C ± 1 °C for subsequent examination in a laboratory located at the El-Sabaheia Research Station, Plant Protection Station, Agriculture Research Center, Alexandria Governorate, Egypt, which is approximately 31.21° N, 29.94° E and 4.45 m above sea level, as indicated in [36]; however, Alexandria Governorate, Egypt, is located at a latitude of 31° 12′56.30″ N and a longitude of 29°57′18.97″ E. The experiment commenced with an initial seed quality assessment within one week of acquisition, for which a representative sample of two hundred randomly selected seeds was collected from the batch for analysis.

### 2.2. Isolation and Purification of Fungi from Rice Seeds

Agar plate methods were used to test rice seed cultivars for the presence of fungi in accordance with ISTA [35]. Potato dextrose agar medium (PDA) was used to sow 200 rice grains with a moisture content of (13.8–14%). Each cultivar was evaluated using eight replications (25 seeds per replication), which were subsequently incubated at 25 °C for five days. After single spore isolation was used to purify the produced fungi, they were sub-cultured on PDA slants and maintained at 4 °C [37]. Based on microscopic analysis and culture morphological traits, the various fungal isolates were identified. For accurate identification, temporary slides were made and examined under a compound light microscope. When feasible, the fungal isolates were identified down to the species level using the relevant keys [38,39,40,41,42,43]. The following formula (Equation (1)) was used to determine the infection percentage:

(1) $$Infection\;precentage\;(\%)=\frac{Number\;of\;seeds\;colonized\;by\;fungus}{Total\;number\;of\;seeds\;studied}\;\times \;100$$

### 2.3. Fungal Identification

After obtaining pure cultures of fungus, their morphological characteristics were examined, and a pure culture of each fungal isolate was identified. Fungi existing on seeds were recognized by employing the explanation sheets of the Commonwealth Mycological Institute (CMI) Kew, Surrey, England, Danish Government Institute of Seed Pathology (DGISP) publications, as reported in Elwakil et al. [44] and Ghoneem et al. [45].

### 2.4. UV-C Rice Sample Treatment

**Figure 1:**
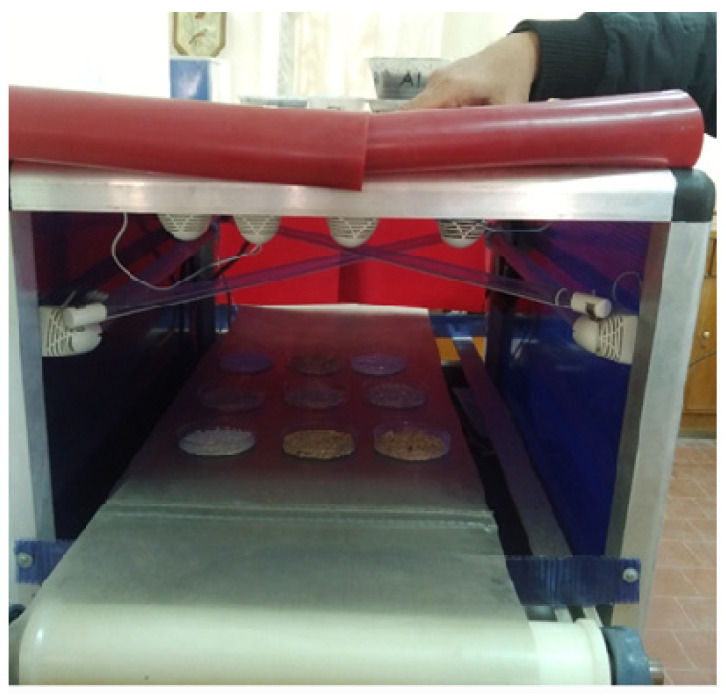
The UV device involved using Petri dishes with rice samples.

A locally manufactured UV-C device developed by El-Maghawry et al. [46] was used to sterilize the rice seed samples in this study. The UV-C treatments were carried out at the Department of Agricultural and Biosystems Engineering, Faculty of Agriculture, Alexandria University, Egypt. The utilized UV-C device (Figure 1), which is described by Morsy et al. [47], involves using Petri dishes with rice samples. Table 1 summarizes the key system components and engineering specifications of the UV-C device.

To shield users from UV-C radiation, the system is completely contained in a fiberglass container. It also has a safety switch that keeps the lights from turning on while the cover is open. For UV-C sterilization, the device can deliver different radiation intensities as reported in Morsy et al.: low intensity, 980 µW/cm^2^ (4 lamps), medium intensity, 1470 µW/cm^2^ (6 lamps), and high intensity, 1960 µW/cm^2^ (8 lamps) [47]. A wavelength of 253.7 nm UV-C was used in the UV-C radiation device during the experiments. The baseline conversion formula to explicitly calculate the dosage for any specific duration in the present study dosage is presented in Equation (2) [48].

Fluence (mJ/cm^2^) = Irradiance (mW/cm^2^) × t (s) (2)

Since 1960 µW/cm^2^ = 1.96 mW/cm^2^, so, Fluence can be determined using Equation (3)

Fluence (mJ/cm^2^) = 1.96 × t (3)

where (t) is the exposure time in seconds.

### 2.5. UV-C Irradiation Effect on Seed Germination and Seedling Growth

As previously mentioned, the UV device was used to administer the UV-C radiation treatment. Treatments included exposure durations of 0 min (control), 10, 20, 30, 40, 50, and 60 min. The seed was put on its own Petri dish. In order to prevent certain seeds from being on top of one another and from being attacked by UV-C radiation, the seeds were positioned during the periods in a way that was as dispersed as possible. The seeds were tested for physiological quality and then sanitary quality after being exposed to varying UV-C radiation dosages.

### 2.6. Experimental Factors, Design, and Germination Procedure

The current experiments were conducted to assess the effects of ultraviolet (UV)-C irradiation on the seed germination percentage, rice seedling growth attributes (height of sprouts and length of primary root for three rice varieties), seed fungal infection percentage, and the specific energy and sterilization cost of the three rice cultivars. The experiment involved two factors: factor 1 comprised three rice varieties (Sakha 105, Sakha 108, and Giza 183) and factor 2 comprised seven UV-C radiation exposure durations (T1 = 0 min, (control), T2 = 10 min, T3 = 20 min, T4 = 30 min, T5 = 40 min, T6 = 50 min, and T7 = 60 min). A randomized complete block design was employed, replicated three times.

**Figure 2:**
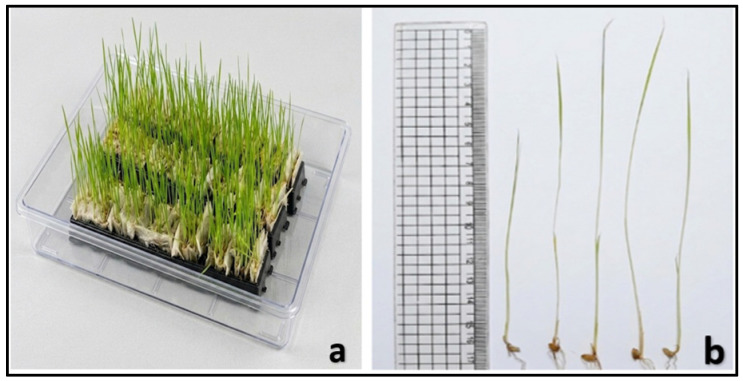
Evaluation of UV-C irradiation on rice seedling and root vigor: (**a**) slide cassette holder units; (**b**) determining the length of the germ and the length of the root after 10 days of treatment.

Rice seed germination was conducted following the ISTA [35] using the slide cassette holder method as developed by Shakya and Chung [49]. The cassette with the filter papers was placed in a tray filled with 200 mL of a solution of urea (230 µg/mL nitrogen), so that 2 cm of the cassette was covered. Once the filter papers became moist, 4 seeds were placed between the two layers of the filter papers. The trays were incubated in high-humidity conditions by putting them under position in a growth room at 28–30 °C under a day/night cycle of 12 h light/12 h darkness. For each variety, 200 seeds per treatment were utilized for the germination tests, with the entire experimental procedure performed in two independent trials to ensure reproducibility. After 3 days (Figure 2), the germination percentage was determined by Equation (4) [4,50,51].

(4) $$Germination\;percentage\;(\%)\;=\;\frac{Number\;of\;germinated\;seeds}{Total\;number\;of\;seeds\;planted}\;\times \;100$$

The length of the roots and shoots of seedlings were measured using a precise ruler with a precision of 1 mm to assess the growth of the seedlings. Ten-day-old seedlings were used to measure early seedling growth characteristics. On the day of the final measurements, 100 seedlings per variety were randomly selected for each treatment and evaluated. The seedlings were carefully taken out, and their shoot and root lengths were measured independently.

### 2.7. UV-C Irradiation Effect on Rice Seed-Borne Fungi

The seeds used in the experiment were counted as 200 seeds for each variety. Rice seeds from each variety were exposed using a UV-C radiation device for 0 (as control), 10, 20, 30, 40, 50, and 60 min. Then, agar plate methods were used to test the effect of UV-C on rice seed-borne fungi compared with the control (not exposed to radiation). The Petri dishes were incubated at 25 °C for five days. After that, the extent of the infection was assessed.

### 2.8. Specific Energy Calculation for the Sterilization Process

The specific energy for the sterilization process using the UV-C device can be calculated as in Equation (5) [47].

(5) $$SE=\frac{Pe\times T\times Ad\times Nt}{Ab\times Qm}$$

where SE is the specific energy for the sterilization process (kW·h/kg), Pe is the lamp power (kW), T is the exposure period (h), Ad is the Petri dish area, (cm^2^), Nt is the number of lamps used (8 lamps), Ab is the belt conveyor area (cm^2^), and Qm is amount of all seeds inside the working place in the UV device (kg).

### 2.9. Cost Calculation of the Sterilization Process

The sterilization process cost when using the UV-C device for rice samples can be calculated using Equation (6) [47].

Sterilization cost (US Dollar/kg) = SE × 0.0285(6)

where SE is the specific energy (kW·h/kg) and the value of 0.0285 is the average price of electricity (US Dollar/kW·h) during the experimental work, and it was used to calculate the cost of a kilowatt-hour of electric power. The average price is based on the rules of the Ministry of Electricity and Renewable Energy in Egypt [52] during the period of the experiment, which was conducted on 16 February 2025; in addition, note that 1 Egyptian pound equals 0.019 United States Dollars (date 10 April 2026).

### 2.10. Statistical and Regression Analysis

A two-way ANOVA technique was conducted using SAS Software v.9.4 to test the significance (*p* < 0.05) of the treatments (exposure duration and rice cultivars) on the seed germination percentage, rice seedling growth rice attributes (height of sprouts and length of primary root), and reduction percentage of seed fungal infection. Least significant difference (LSD) at a 5% significance level was used to compare the arithmetic means of the variables.

The regression technique, which is statistical method employed for model fitting that aims to find a relationship between variables and frequently used linear or polynomial approaches to minimize the sum of squared errors between model predictions and observed data [53], is used in this study. The association between germination percentage, sprout height, primary root length, and exposure period for each rice cultivar was examined in this study utilizing a variety of basic regression models using an Excel spreadsheet. The regression model with the highest coefficient of determination (R^2^) was chosen.

## 3. Results

### 3.1. Determining the Seed-Borne Fungi of the Three Varieties of Egyptian Rice

The screening of seed-borne fungi in the three Egyptian rice cultivars (Sakha 105, Sakha 108, and Giza 183) discovered significant variations in fungal distribution:

- Dominant pathogens: *Alternaria alternata* emerged as the most frequent isolate, showing its highest prevalence in Giza 183 (32%), followed by Sakha 105 and Sakha 108 (16% each), as shown in Table 2
- Cultivar susceptibility: A high incidence of *Rhizoctonia solani* was specifically noted in Sakha 108 (20%), whereas *Drechslera oryzae* and high levels of *Fusarium verticillioides* (16%) were primarily associated with Sakha 105.
- Storage fungi: *Aspergillus* and *Penicillium* species showed higher frequency in Sakha 105, while Sakha 108 appeared more resistant to these specific contaminants.

### 3.2. Statistical and Regression Analysis of the Impact of Ultraviolet (UV-C (Radiation Exposure Duration on the Rice Seed Germination Percentage, Height of Sprouts, Length of Primary Root, and Reduction Percentage of Seed Fungal Infection for the Three Rice Varieties

The UV-C radiation exposure duration significantly (*p* < 0.05) affected the rice seed germination percentage, height of sprouts, length of primary root, and reduction percentage of seed fungal infection of the investigated rice varieties (Table 3). Treatment T4 (30 min exposure duration) led to the highest seed germination percentage (99.94%), sprout height (16.4 cm), primary root length (14.0 cm), and reduction in the percentage of seed fungal infection (81.33%) (Table 4). The lowest seed germination percentage, height of sprouts, length of primary root, and reduction percentage of seed fungal infection belonged to the control exposure duration (T1, 0 min).

While the percentage of broad-spectrum seed fungal infection was monitored comprehensively over all exposure intervals (T0 to T7, as given in Table 4), specific isolated colony profiling for individual fungal species (*Alternaria alternata*, *Rhizoctonia solani*, and *Fusarium verticillioides*) was limited to the 30 min treatment (T4). This particular interval was selected for further taxonomic screening in view of the fact that our main agronomic data indicated T4 as the last physiological turning point, which maximized germination vigor, with a drastic and statistically significant reduction in the total amount of fungi with respect to the control (T1). Therefore, the kinetics of individual species over the intermediate 15, 45, and 60 min thresholds are beyond the scope of this deep profile, but the total infection kinetics validated in Table 4 provide the baseline proxy, validating T4 as the operational optimum.

Table 5 indicates the effect of rice varieties on the mean of seed germination percentage, height of sprouts, length of primary root, and reduction percentage of seed fungal infection. As shown, the trend of investigated attributes had different behaviors. For the seed germination percentage, Giza 183, when treated with UV-C, had the highest mean percentage (92.40%) regarding the height of sprouts and the length of primary root. The Sakha 105 variety, when treated with the UV-C radiation, had the highest mean height of sprouts (17.00 cm) and the highest length of primary root (12.91 cm). For the reduction percentage of seed fungal infection, the Sakha 108 variety, when treated with UV-C radiation, had the highest mean value (58.86%), as depicted in Table 4. Together, the results indicate that cultivar-dependent differences were expressed across various assessed variables, emphasizing the relevance of varietal traits in defining the overall response reported in this study.

**Figure 3:**
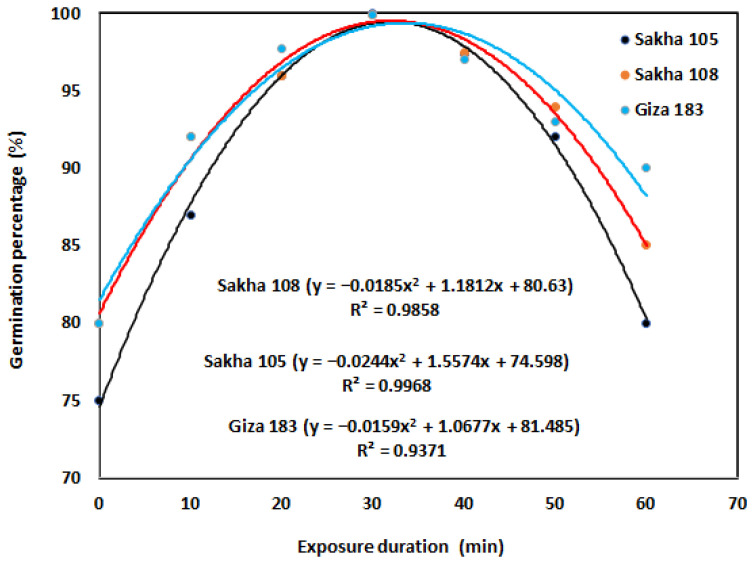
The effect of UV-C exposure duration on germination percentage of three rice varieties.

**Figure 4:**
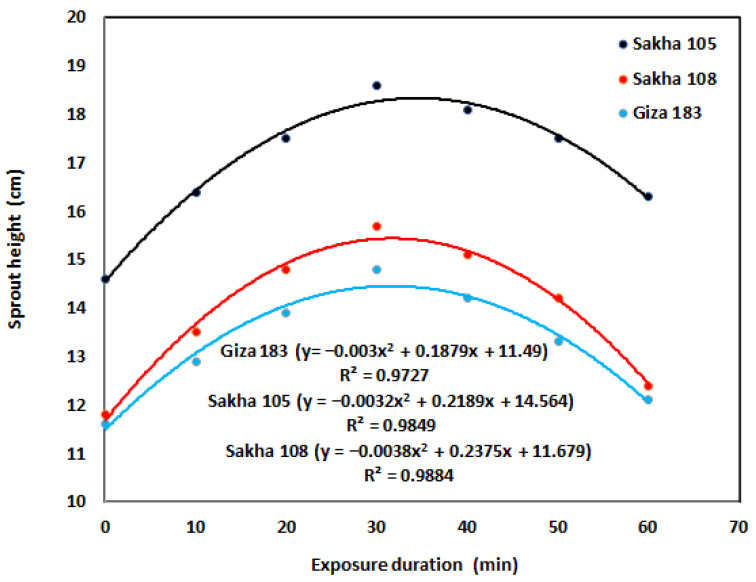
The effect of UV-C radiation exposure duration on the sprout height for three rice varieties.

**Figure 5:**
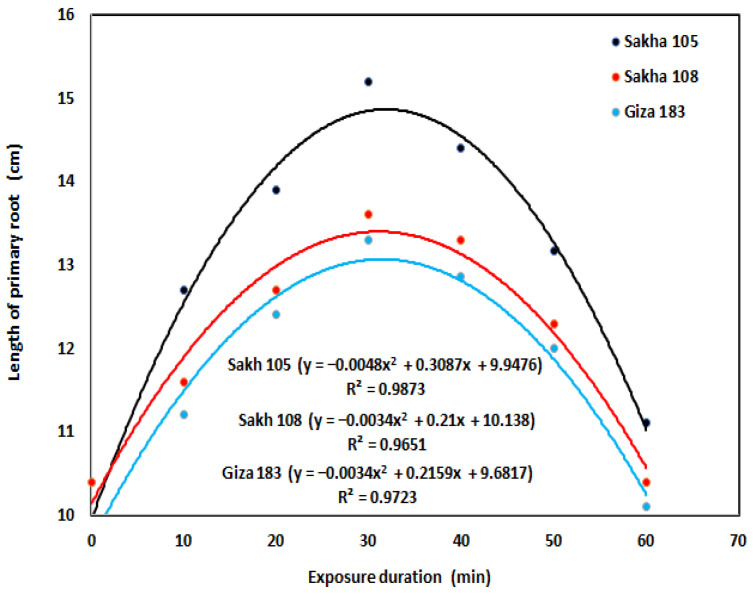
The effect of UV-C radiation exposure duration on the length of primary root for the three rice varieties.

Regression analysis revealed that the physiological responses of rice seed cultivars Sakha 105, Sakha 108, and Giza 183 to the exposure duration to UV-C were quadratic in nature for germination percentage, sprout height, and primary root length (Figure 3, Figure 4 and Figure 5). However, the points shown in Figure 3, Figure 4 and Figure 5 represent the average of the observed values. The high coefficients of determination for germination percentage (R^2^ = 0.9373–0.9968), sprout height (R^2^ = 0.9727–0.9884) and primary root length (R^2^ = 0.9651–0.9873) confirmed that the quadratic models can accurately describe the variation in these traits and determine the optimum exposure threshold. The best physiological response was obtained after 30 min of UV-C exposure in all cultivars, with Sakha 105 having the highest germination percentage (99.97%), sprout height (18.7 cm) and primary root length (15.2 cm). However, the exposure duration was increased to 60 min, which resulted in a remarkable reduction in all the assessed traits and indicated that long exposure to UV-C was inhibitory to the germination of rice seeds and early seedling growth.

To calculate the optimum UV-C exposure time from a quadratic regression equation, we used the vertex formula:

x = −b/2a(7)

where x is the UV-C exposure time in min.

The quadratic equation is

y = ax^2^ + bx + c

In this study and based on equation 7, Table 6 indicates the answering UV-C exposure time and answering responses (germination percentage, sprout height, and primary root length) for the three investigated rice cultivars.

The answering UV-C exposure times for the tested responses ranged from 30.88 to 33.58 min according to the analytical analysis, indicating that the experimentally chosen exposure period of 30 min was around the analytical answering. 

### 3.3. Impact of 30 Min UV Exposure Duration on Rice Seed Fungal Infection Rate

**Figure 6:**
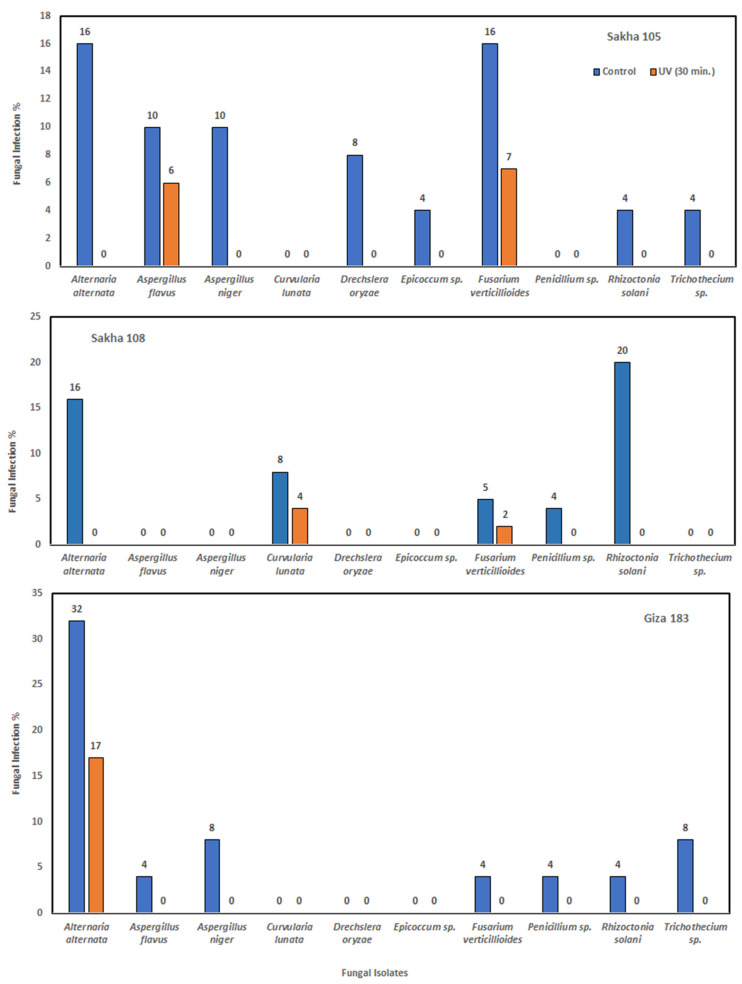
Effect of rice seed treatment by UV–C radiation on fungal infection percentage growth in vitro.

Because it offers a practical and efficient method for inactivating harmful microorganisms, sterilization by exposure to UV radiation is gaining special interest [54]. In the experimental work, the UV-C treatments applied to the three varieties of rice (Figure 6) showed effects toward infection reduction after seven days of incubation and exposure time (30 min) with UV-C irradiance.

The results in Figure 6 reveal that the exposure time of 30 min led to a substantial reduction in or total elimination of the associated mycoflora across all cultivars. In Giza 183, the dominant pathogen *Alternaria alternata* was reduced from a control level of 32% to approximately 17%, while other associated fungi such as *Aspergillus niger*, *A. flavus*, *Penicillium* sp., and *Trichothecium* sp. were completely eradicated. For Sakha 108, the treatment was exceptionally effective, eliminating the high initial infection by *Rhizoctonia solani* (20% in control) and *Curvularia lunata*, leaving only a trace presence of *F. verticillioides*. UV-C irradiation was effective in eliminating *Alternaria alternata*, *Aspergillus niger* and *Drechslera oryzae* in Sakha 105 but *F. verticillioides* and *A. flavus* were present at a lower level than the control. The data presented here show that UV-C irradiation for 30 min is an effective way of dealing with a broad spectrum of diseases, thus significantly improving the phytosanitary quality of seeds.

### 3.4. Effect of Using UV-C for Different Exposure Times on Specific Energy for the Sterilization Process and Sterilization Cost of Rice Cultivars

Table 7 shows the effect of UV-C exposure time on the specific energy consumption for sterilization and sterilization cost for rice seeds. Both parameters increase gradually with increasing exposure times, showing the direct proportionality between the treatment time and energy input. At lower exposure times (10–20 min) the values are relatively low, indicating less energy demand and lower sterilization costs. The specific energy (kW·h/kg) and sterilization cost (US Dollar/kg) shown in Table 6 are calculated on the bases of the experimental data.

Although the 30 min treatment showed moderate increments of specific energy consumption for the sterilization process and sterilization cost, the results are in agreement with previous findings that this is the optimal duration for germination improvement and reduction in fungi incidence, as well as an efficient compromise between efficiency and cost. In contrast, the increases in specific energy consumption for the sterilization process and sterilization cost were more relevant for treatments beyond 40 min, with the highest values being at 60 min, indicating a deteriorating economic viability with longer exposure time, particularly as longer treatments were also associated with lower seedling vigor. The data in Table 6 confirms that moderate UV-C exposure (around 30 min) can achieve effective sterilization with acceptable specific energy consumption for the sterilization process and sterilization cost, while longer durations imply higher economic and biological penalties.

## 4. Discussion

The results of the present study showed that UV-C irradiation might be considered a viable non-chemical and eco-friendly strategy for seed-borne pathogen disinfection and early growth performance of Egyptian rice cultivars. The preliminary screening revealed the high incidence of basal fungal infection with the prevalence of *Alternaria alternata* (32.0%), *Rhizoctonia solani* (20.0%), and *Fusarium verticillioides* (16.0%).

Morphological characterization was the main method for fungal identification in the present study. Sequence-based characterization is the gold standard for definitive diagnostics, but a rigorous morphology-based approach using CMI taxonomic keys was systematically adopted to suit the large pool of isolates obtained from three different rice cultivars under several UV-C exposure times. Given this large sample size, classical taxonomy provided a highly viable approach that directly corresponds to the main objective of the study. However, incorporating molecular validation is still a necessary prospective step to achieve deeper taxonomic resolution in future investigations.

It is interesting to note that all examined cultivars (Sakha 105, Sakha 108, and Giza 183) showed a distinct biphasic response over time when treated with UV-C radiation. The time point of 30 min was the important threshold leading to the highest seed germination (up to 99.97% in Sakha 105) as well as the largest sprout height and main root length. This time point also offered a good compromise of fungal suppression without the seed viability regressions seen at the 40 and 60 min exposure intervals. Furthermore, the quadratic regression modeling accurately predicted these germination thresholds, and the economic analysis demonstrated that this optimal 30 min treatment is highly cost-effective under local electricity pricing. These results thus suggest that UV-C treatment is highly successful in reducing fungal infection and promoting growth, but its efficacy is rigidly dictated by exact exposure thresholds and cultivar-specific sensitivity.

UV seed irradiation relies on the technique of pre-sowing photostimulation influenced by sun radiation, a practice familiar to our forebears, as only artificial UV radiation is utilized instead of sunlight. As a non-thermal and residue-free technology, UV-C irradiation serves as a robust broad-spectrum disinfectant that effectively suppresses diverse microbial pathogens. Controlling exposure to UV radiation has a positive effect on the biological resistance of plants. Apart from sanitation, controlled exposure stimulates systemic resistance and improves biological resilience in various agricultural applications. Exposure of seeds to ultraviolet light causes activation of the latent biological reserves of plants, which results in better germination and germination intensity. In addition, UV-C radiation is used for the disinfection of germs. It is well known that the microbial community of plants, including rice, is greatly influenced by a variety of environmental factors. Specifically, as rice is a food crop that needs to be both extremely productive and nutritious [1,5,18,55], the most intriguing subject is the UV-C treatment of rice seeds, which has garnered a lot of interest due to its impact on the host plant’s growth and productivity in addition to the seeds’ quality and safety.

One of the prerequisites for high-quality seed criteria is pathogen-free seed. In our investigation, we found a number of fungi associated with three varieties of local rice seeds in Egypt. The percentage of infection varies with different varieties, but it was found that *Alternaria alternata*, *Rhizoctonia solani*, and *Fusarium verticillioides* were present in all three varieties. The remaining fungi were found infrequently. Additionally, since they most likely have no pathological significance, additional saprophytic taxa of related fungi are not taken into further consideration. The results were consistent with those of [23,56,57,58].

The results of the study show that the length of exposure determines how UV-C radiation affects the germination and growth of three different varieties of rice. Rice seed germination was observed to benefit from moderate UV-C radiation exposure for 30 min. In comparison to the control group, these exposure times produced noticeably greater germination percentages, faster germination times, and enhanced germination indices.

UV radiation (such as UV-B and UV-C) provides high-energy photons that physically weaken the seed coat. This allows the seed to imbibe water and oxygen much faster [4]. The enhanced germination performance of seedlings exposed to UV-C light is explained by its capacity to cause the seed coat to accumulate advantageous secondary metabolites like flavonoids and saponins, as well as elevated reactive oxygen species (ROS) concentrations and a rise in temperature following UV-C radiation exposure [31,59]. The seeds can be shielded from a number of environmental challenges, including oxidative stress, pathogen attack, and UV radiation itself, by this build-up of advantageous secondary metabolites. Furthermore, ROS produced by UV-C exposure might cause adaptive reactions in seeds, encouraging greater oxygen and water imbibition and strengthening their resistance to stresses.

The antioxidant pathway is also important for the development of stress tolerance and the increase in antioxidant enzymes such as peroxidase, catalase and polyphenol oxidase as a result of the development of defense mechanisms against oxidative stress. However, the decrease in the enzyme activity at higher UV doses of more than 25–30 min may be due to the stress of prolonged UV exposure that can have a catastrophic effect on cellular structures [16]. In one soybean variety, [60] found that the UV treatment improved seed germination in the laboratory. In addition, the specific activity of guaiacol peroxidase and ascorbate oxidase changed, demonstrating the interaction of antioxidant system components in the seeds and proposing the emergence of adaptive mechanisms. Inayat et al. [61] claimed that UV radiation induces an intensity-dependent response in radish plants: the separation of beneficial adaptation from oxidative stress. At low levels of UV light the plant can safely acclimate; however, at high exposure the plant’s growth is significantly reduced, and crucial photosynthetic processes are reduced by over 60%. To combat this damage the plant extensively activates oxidative protective enzymes (such as superoxide dismutase and catalase), diverting energy from growth and devoting it entirely to defense. However, such mechanisms were not measured in this study and can be presented only as possible explanations. Additionally, these mechanisms provide a speculative framework to interpret the observed results. They offer potential pathways to explain the data.

On the other hand, rice seeds started to suffer when exposed to UV-C light for extended periods of time, such as 60 min. The germination percentage decreased significantly throughout these exposure times. Increased cellular damage could be the reason for the lack of growth response at larger doses or stress reactions that surpass the advantages of adaptation. Antioxidant mechanisms may be activated as a result of the massive rise in ROS, which might shift resources from development activities to defense responses. The increased activity of antioxidant enzymes like catalase, which helps minimize oxidative damage but may also lead to slower growth rates, is indicative of this change [62,63]. According to Çavuşoğlu et al. [64], UV-C radiation exposure caused cytogenotoxicity, growth inhibition, and damage to the roots’ meristematic cells. The type of radiation, the wavelength, and the length of exposure all play a role in the effectiveness of the treatment.

Infection percentage in rice seeds is greatly inhibited by UV-C irradiation, as evidenced by the result that all varieties’ mean fungal incidence dropped following the treatment. Sakha 108 had the greatest decline. The fungicidal action that damages microbial DNA may be the cause of this impact [15]. This technique effectively disinfects the surface of seeds without affecting their germination rates when applied at the right doses. However, variety Giza183 was found to be less impacted by UV treatment than the other two varieties. This could be because in rice plants, including seedlings and growing seeds, phenolic chemicals serve as a natural sunscreen and antioxidant and are the main defense mechanism against UV radiation. These substances are found in high concentrations in the bran and husk layer of rice seeds [63]. Furthermore, phenolic compounds neutralize ROS and lessen cellular damage by acting as strong antioxidants and free radical scavengers. Momilactones, a kind of diterpenoid also present in rice, and phenolics, such as salicylic acid, may function as signaling molecules, initiating a series of defense reactions and boosting the plant’s general resistance to UV stress [65]. In the present study, the role of ROS can be proposed only as a possible explanation for the observed changes in the response of fungi to UV-C irradiation. Similarly, phenolic compounds and related defense metabolites can participate in stress adaptation and antimicrobial resistance in plant–pathogen systems, but their participation in the present experiment is indirect, unless supported by biochemical measurements.

The Giza 183 variety was released in 2023 in Egypt as a new rice variety to resist biotic and abiotic stresses. Genetic diversity analysis revealed that Giza 183 belongs to the indica/japonica type [66]. On the other hand, Sakha 108 rice is primarily classified within the japonica group [67]. Compared to indica rice, japonica rice often has a greater overall content of phenolic compounds and antioxidant activity, especially in the bran and husk layers. Although the precise phenolic compounds and their amounts can change among varieties within each category, the levels of these compounds are frequently higher in japonica rice. Additionally, even among varieties of the same kind, the precise concentrations of phenolics, such as p-hydroxybenzonic acid, can vary considerably [10,68,69]. This notion is intriguing; however, it should be clearly mentioned as a testable hypothesis for future work because it was found that Giza 183 was less susceptible to UV-C due to greater phenolic content due to indica/japonica genetics, which was not tested in this study.

In terms of the UV treatment’s impact on reducing the infection rate, it was found that *Alternaria alternata*, *Curvularia lunata*, and *A. flavus* were not completely inhibited. This may be because fungi have evolved a number of defense mechanisms against UV light. This covers both DNA repair mechanisms and direct defense mechanisms like pigment production [70,71].

By combining biological performance (germination and growth), phytosanitary impacts (fungal suppression), and techno-economic analysis (energy consumption and cost), this study offers a thorough assessment of UV-C irradiation as a dual-function seed treatment technology for rice. In contrast to earlier research, this work provides a workable and scalable strategy for sustainable seed treatment by determining the ideal exposure length (30 min) based on both physiological and economic parameters. From an applied standpoint, UV-C treatment is a viable, eco-friendly method for disinfecting seeds in rice production systems. In line with sustainable agricultural methods, it lessens dependency on chemical fungicides.

### Limitations and Future Research Directions

One structural boundary of our phytosanitary evaluation framework should be highlighted. While the main agronomic traits and the whole seed fungal infection percentages were systematically mapped over the entire spectrum of exposures (0 to 60 min), a detailed taxonomic species profiling was restricted to the optimized threshold of 30 min. Although this targeted characterization validates the effectiveness of a UV-C dose of 3528 J/cm^2^ for the suppression of the dominant seed-borne pathogens (*A. alternata*, *R. solani*, and *F. verticillioides*) without negative impacts on seed viability, it limits our capability to build explicit, independent degradation curves for each individual fungal species at intermediate intervals. Future studies should expand this screening to capture species-specific inactivation kinetics at all exposure durations. This would confirm whether individual pathogens present unique sensitivity thresholds, thereby further refining targeted seed-sanitation. Further research is needed to elucidate the extent of benefits of this approach under large-scale and field conditions, particularly in view of factors such as seed lot variability, equipment set-up and climatic influences. Consequently, field validation, storage evaluation and large-scale seed treatment trials are still needed.

## 5. Conclusions

In some rice cultivars, this study showed that UV-C radiation is an efficient physical method for lowering fungal contamination and influencing seed germination and early seedling growth. The findings demonstrated that treatment outcomes are significantly influenced by the length of UV-C exposure. High exposure was harmful to the germination and development of seedlings, probably due to cellular and physiological damage, whereas moderate exposure resulted in a positive balance between suppression of the fungus and viability of the seed. Responses were also cultivar-dependent, indicating that different genotypes of rice have different levels of tolerance to UV-C radiation. This study emphasizes how crucial it is to tailor treatment settings for particular cultivars rather than using a standard exposure protocol. All things considered, UV-C treatment exhibits great promise as a long-term substitute for chemical fungicides in seed treatments. However, better disinfection efficacy and fewer adverse effects on seed germination require strict regulation of exposure time in the practical application of this method. Further research should be directed at extending the treatment regime, studying the effects of prolonged storage and testing the method under field conditions. Furthermore, the physiological and biochemical mechanisms of seed responses to UV-C irradiation need to be further studied.

## Figures and Tables

**Table 1:** Key system components and engineering specifications.

Component/Parameter	Detailed Technical Specification
System housing	Enclosed steel cabinet outfitted with an internal light-impermeable acrylic chamber and an outer protective fiberglass containment shell structure.
Overall dimensions (cm)	71.0 cm × 51.0 cm × 35.5 cm (length × width × height). Internal dimensions denote maximum object volumetric limits.
UV-C radiation lamps	8 Philips 83W germicidal lamps (Model: LTC80T5/4 UV-C Germicidal Lamp) configured in an overhead multi-lamp array cluster.
Lamp wavelength	Peak germicidal output at 253.7 nm within the short-wave UV-C spectrum.
Radiation intensity	245 µw/cm^2^ steady-state radiant density measured at target belt surface area/per lamp cluster zone.
Service life	Performance threshold rated at 100 operating hours.
Surface areas	• Conveyor belt (max. usable treatment area): up to 0.24 m^2^. • Standard sample Petri dish (90mm): 0.00636 m^2^ per dish.
Safety feature	Integrated automatic micro-interlock cover safety switch. Completely isolates and disables power supply to prevent accidental radiation exposure when the chamber cover is opened.

**Table 2:** Percentage of seed-borne fungi with three local Egyptian rice varieties for 200 seeds of each variety.

Fungi Species	Sakha 105	Sakha 108	Giza 183
*Alternaria alternate*	16	16	32
*Aspergillus flavus*	10	0	4
*Aspergillus niger*	10	0	8
*Curvularia lunata*	0	8	0
*Drechslera oryzae*	8	0	0
*Epicoccum* sp.	4	0	0
*Fusarium verticillioides*	16	5	4
*Penicillium* sp.	0	4	4
*Rhizoctonia solani*	4	20	4
*Trichothecium* sp.	4	0	8

**Table 3:** Analysis of variance (ANOVA, *p* < 0.05) results of the effect of the UV-C radiation exposure duration and rice varieties on the investigated parameters (Df is degree of freedom).

Source of Variation	Df	F Value	Pr>F	F Value	Pr>F	F Value	Pr>F	F Value	Pr>F
		Germination Percentage		Height of Sprouts		Length of Primary Root		Reduction Percentage of Seed Fungal Infection	
Replicates	2	84.66	<0.001	1.42 × 10^16^	<0.001	6.17	0.0046	12.31	<0.001
Exposure length (DL)	6	389.66	<0.001	1.14 × 10^17^	<0.001	199.07	<0.001	1413.01	<0.001
Rice varieties (V)	2	43.46	<0.001	6.28 × 10^17^	<0.001	83.44	<0.001	185.38	<0.001
DL × V	12	8.99	<0.001	1.67 × 10^15^	<0.001	4.16	0.0003	9.82	<0.001

**Table 4:** Comparison of the arithmetic means * of exposure duration based on seed germination percentage, height of sprouts, length of primary root, and reduction percentage of seed fungal infection.

Exposure Duration	Rice Seed Germination Percentage (%)	Height of Sprouts (cm)	Length of Primary Root (cm)	Reduction Percentage of Seed Fungal Infection (%)
T1 = 0 min, control	78.33 g	12.67 g	10.07 g	0 g
the T2 = 10 min	90.33 e	14.27 e	11.83 e	38.07 f
T3 = 20 min	96.57 c	15.40 c	13.00 c	65.00 cd
T4 = 30 min	99.94 a	16.37 a	14.03 a	81.33 a
T5 = 40 min	97.27 b	15.80 b	13.52 b	75.00 b
T6 = 50 min	93.00 d	15.00 d	12.49 d	65.33 dc
T7 = 60 min	85.00 f	13.60 f	10.53 f	41.67 f
LSD (5%)	1.105	11 × 10^−9^	0.3013	2.1334

* Different letters within a column show a statistically significant difference.

**Table 5:** Comparison of the arithmetic means * of the rice varieties treated with UV-C based on seed germination percentage, the height of sprouts, the length of primary root, and the reduction percentage of seed fungal infection.

Rice Varieties	Rice Seed Germination Percentage (%)	Height of Sprouts (cm)	Length of Primary Root (cm)	Reduction Percentage of Seed Fungal Infection (%)
Giza 183	92.40 a	13.26 c	11.68 c	45.57 c
Sakha 108	92.06 b	13.93 b	12.04 b	58.86 a
Sakha 105	89.61 c	17.00 a	12.91 a	52.86 b
LSD (5%)	0.7234	72 × 10^−10^	0.1972	1.3967

* Different letters within a column show a statistically significant difference.

**Table 6:** The answering UV-C exposure time and answering responses (germination percentage, sprout height, and primary root length) for the three investigated rice cultivars.

Rice Cultivar	Response	The Quadratic Equation	Answering Time From Equation (7) (min)	Answering Response
Sakha 105	Germination percentage (%)	y = −0.0244 × ^2^ + 1.5574x + 74.598	31.91	99.45%
	Sprout height (cm)	y = −0.0032 + 0.2189x + 14.564	34.20	18.31 cm
	Length of primary root (cm)	y = −0.0048 + 0.3087x + 9.9476	32.16	14.91 cm
Sakha 108	Germination percentage (%)	y = −0.0185 × ^2^ + 1.1812x + 80.63	31.92	99.48%
	Sprout height (cm)	y = −0.0038 × ^2^ + 0.2375x + 11.679	31.25	15.39 cm
	Length of primary root (cm)	y = −0.0034 × ^2^ + 0.21x + 10.138	30.88	13.38cm
Giza 183	Germination percentage (%)	y = −0.0159 × ^2^ + 1.0677x + 81.485	33.58	99.41%
	Sprout height (cm)	y = −0.003 × ^2^ + 0.1879x + 11.49	31.32	14.43 cm
	Length of primary root (cm)	y = −0.0034 × ^2^ + 0.2159x + 9.6817	31.75	13.11 cm

**Table 7:** Effect of using UV-C for different exposure times on specific energy and sterilization cost of rice cultivars.

Exposure Duration	Specific Energy (kW.h/kg)	Sterilization Cost (US Dollar/kg)
10 min	0.0049	0.000137
20 min	0.0098	0.000276
30 min	0.0147	0.000416
40 min	0.0195	0.000555
50 min	0.0244	0.000695
60 min	0.0293	0.000834

## Data Availability

The original contributions presented in this study are included in the article. Further inquiries can be directed to the corresponding authors.
